# Early Referral to a Nephrologist Improved Patient Survival: Prospective Cohort Study for End-Stage Renal Disease in Korea

**DOI:** 10.1371/journal.pone.0055323

**Published:** 2013-01-25

**Authors:** Do Hyoung Kim, Myounghee Kim, Ho Kim, Yong-Lim Kim, Shin-Wook Kang, Chul Woo Yang, Nam-Ho Kim, Yon Su Kim, Jung Pyo Lee

**Affiliations:** 1 Department of Internal Medicine, Seoul National University College of Medicine, Seoul, Korea; 2 Department of Dental Hygiene, College of Health Science, Eulji University, Seongnam, Korea; 3 Department of Epidemiology and Biostatistics, School of Public Health, Seoul National University, Seoul, Korea; 4 Department of Internal Medicine, Kyungpook National University School of Medicine, Daegu, Korea; 5 Department of Internal Medicine, Yonsei University College of Medicine, Seoul, Korea; 6 Department of Internal Medicine, The Catholic University of Korea College of Medicine, Seoul, Korea; 7 Department of Internal Medicine, Chonnam National University Medical School, Gwangju, Korea; 8 Department of Internal Medicine, Seoul National University Boramae Medical Center, Seoul, Korea; 9 Clinical Research Center for End Stage Renal Disease in Korea, Daegu, Korea; Sookmyung Women's University, Republic of Korea

## Abstract

The timing of referral to a nephrologist may influence the outcome of chronic kidney disease patients, but its impact has not been evaluated thoroughly. The results of a recent study showing an association between early referral and patient survival are still being debated. A total of 1028 patients newly diagnosed as end-stage renal disease (ESRD) from July 2008 to October 2011 were enrolled. Early referral (ER) was defined as patients meeting with a nephrologist more than a year before dialysis and dialysis education were provided, and all others were considered late referral (LR). The relationship of referral pattern with mortality in ESRD patients was explored using a Cox proportional hazards regression models. Time from referral to dialysis was significantly longer in 599 ER patients than in 429 LR patients (62.3±58.9 versus 2.9±3.4 months, P<0.001). Emergency HD using a temporary vascular catheter was required in 485 (47.2%) out of all patients and in 262 (43.7%) of ER compared with 223 (52.0%) of LR (P = 0.009). After 2 years of follow-up, the survival rate in ER was better than that in LR (hazard ratio [HR] 2.38, 95% confidence interval [CI] 1.27–4.45, P = 0.007). In patients with diabetes nephropathy, patient survival was also significantly higher in ER than in LR (HR 4.74, 95% CI 1.73–13.00, P = 0.002). With increasing age, HR also increased. Timely referral to a nephrologist in the predialytic stage is associated with reduced mortality.

## Introduction

Patients with end-stage renal disease (ESRD) have an exceedingly high morbidity and mortality compared to the general population [1]. The number of ESRD patients is growing at a much faster rate than the total population. The increasing prevalence of ESRD has led to its recognition as a significant clinical and public health problem, in terms of its use of medical resources and public health expenditure. According to data from the National Health Insurance Corporation, chronic kidney disease (CKD) ranked first in public health expenditure [2]. Also, it was the single most expensive disease.

Over the past several years, interest has evolved in evaluating the timing of nephrology referral in the predialytic stage of CKD. The potential benefits of timely nephrology referral include identification of reversible causes of CKD, provision of treatments that may slow the progression of CKD, management of the metabolic complications of advanced CKD, coordination of education regarding ESRD treatment options, and optimal preparation for the chosen dialysis modality or kidney transplantation [3]. Therefore, delayed nephrology care could be associated with several unfavorable outcomes, including reduced access to peritoneal dialysis (PD) [4], [5] and kidney transplantation [6], [7], higher rates of dialysis initiation through a temporary venous catheter, and an higher mortality rate after starting maintenance dialysis, especially during the first few months [8]–[10].

To date, most referral studies have had a retrospective design [4], [5], [11]–[13] and there have been few prospective multi-center studies examining referral practices [14], [15]. The results of a recent study showing an association between early referral and patient survival are still being debated [16]. Additionally, late referral was defined as the first encounter with a nephrologist occurring within 1 to 3 months of the commencement of dialysis in most studies. This duration was too short to ensure effective education of the patients by the nephrologist [17]. Therefore, a large prospective study is required to investigate the relationship between early referral to a nephrologist and patient survival.

The purpose of this study was to explore the impact of early nephrology referral and frequent attendance at nephrology clinics before ESRD treatment initiation on patient survival in a subset of patients from the Comprehensive Prospective Study of the Clinical Research Center for End Stage Renal Disease (CRC ESRD) in Korea.

## Subjects and Methods

### Study Design and Definition

This study was a multi-center, prospective cohort study of patients with ESRD in Korea who were initiated on dialysis therapy. Patients were divided into two groups according to timing of referral to a nephrologist.

Patients were classified as early referral (ER) if their first encounter with a nephrologist occurred more than 1 year prior to initiation of dialysis and education about dialysis (from a nurse or nephrologist), and all others were considered late referral (LR), as described previously by Di Napoli *et al*. [18].

Estimated glomerular filtration rate (eGFR) was calculated by the modified Modification of Diet in Renal Disease equation as follows: eGFR (mL/min/1.73 m^2^)  = 186.3× (serum creatinine)^−1.154^× (age)^−0.203^ (×0.742 for females) where serum creatinine is measured in milligrams per deciliter and age is measured in years [19].

### Study Population

Patients aged 20 years or more with ESRD who were initiated on dialysis were enrolled in the CRC ESRD. It was a nationwide web-based multi-center joint network prospective cohort of patients with ESRD in Korea designed to improve survival rates and quality of life in patients with ESRD and to create effective treatment guidelines (clinicaltrial.gov NCT00931970). Thirty-one hospitals and clinics in Korea participated in the CRC ESRD and shared the clinical data of 1,211 newly diagnosed adult ESRD patients from July 2008 to October 2011. In the present study 1,028 patients were enrolled among them. Of the 183 patients who were excluded, data regarding visits to a nephrologist were unknown in 133 patients and data regarding the type of initial dialysis were unknown in 50 patients. All patients provided their written consent to participate in this study. All traceable identifiers were removed before analysis to protect patient confidentiality. The study was approved by the institutional review board at each center [The Catholic University of Korea, Bucheon St. Mary's Hospital; The Catholic University of Korea, Incheon St. Mary's Hospital; The Catholic University of Korea, Seoul St. Mary's Hospital; The Catholic University of Korea, St. Mary's Hospital; The Catholic University of Korea, St. Vincent's Hospital; The Catholic University of Korea, Uijeongbu St. Mary's Hospital; Cheju Halla General Hospital; Chonbuk National University Hospital; Chonnam National University Hospital; Chung-Ang University Medical Center; Chungbuk National University Hospital; Chungnam National University Hospital; Dong-A University Medical Center; Ehwa Womens University Medical Center; Fatima Hospital, Daegu; Gachon University Gil Medical Center; Inje University Pusan Paik Hospital; Kyungpook National University Hospital; Kwandong University College of Medicine, Myongji Hospital; National Health Insurance Corporation Ilsan Hospital; National Medical Center; Pusan National University Hospital; Samsung Medical Center, Seoul; Seoul Metropolitan Government, Seoul National University, Boramae Medical Center; Seoul National University Hospital; Seoul National University, Bundang Hospital; Yeungnam University Medical Center; Yonsei University, Severance Hospital; Yonsei University, Gangnam Severance Hospital; Ulsan University Hospital; Wonju Christian Hospital (in alphabetical order)]. All clinical investigations were conducted in accordance with the guidelines of the 2008 Declaration of Helsinki.

### Outcomes

The primary outcome of interest was all-cause mortality after initiation of dialysis in the LR versus ER patients. The secondary outcomes consisted of various clinical and laboratory parameters in the LR versus ER group: emergency hemodialysis (HD), cardiovascular death, cause of death, hospitalization.

### Data Sources

CRC ESRD served as the primary source of data for these analyses. Clinical and laboratory data were collected by web-based medical and patient questionnaires [20].

Each centers collected the information of several outcomes including cause of death with the clinical and laboratory data in this study. Then, they reported to CRC-ESRD web-based registry about the outcomes. Research coordinators from central center carried out a regular sample survey on enrolled patients to confirm the medical records twice a year. They had checked all the medical records of patients who died in hospital registered in CRC-ESRD to confirm the cause-specific death and the mortality date. In the case of patient death in other hospitals, information of cause-specific death was extracted from the Korean National Statistical Office data as of December 31, 2010.

A medical questionnaire was completed by data coordinators, who were trained in each center to collect patient data by a combination of chart reviews and informal interviews using a standardized form. The questionnaires included data on demographics, previous medical history, laboratory results, dialysis modality and prescription, presence and type of permanent access used for the first dialysis, and medications including erythropoietin use. eGFR, modified Charlson co-morbidity index (CCI), and Davies co-morbidity index (DCI) at the initiation of dialysis were recorded for each patient.

The modified CCI and DCI values were calculated using the method described in a previous study [21]–[23]. The modified CCI includes age (weight 1 for every 10 years starting from 50 years of age) and contains 19 categories of comorbidities including congestive heart failure (weight 1), myocardial infarction (weight 1), chronic pulmonary disease (weight 1), cerebrovascular disease (weight 1), hemiplegia or paraplegia (weight 2), dementia (weight 1), diabetes (weight 1), diabetes with complications (weight 2), leukemia (weight 2), lymphoma (weight 2), malignancy (weight 2), metastatic solid tumor (weight 6), mild liver disease (weight 1), moderate or severe liver disease (weight 3), peptic ulcer disease (weight 1), peripheral vascular disease (weight 1), rheumatologic disease (weight 1), renal disease (weight 2) and acquired immune deficiency syndrome (weight 6) [21], [22]. The DCI includes malignancy (1 point), ischemic heart disease (1 point), peripheral vascular disease (1 point), left ventricular dysfunction (1 point), diabetes mellitus (1 point), systemic collagen vascular disease (1 point), and other significant pathology (1 point). These data were obtained from a systematic review of all available records [23].

Patients who responded to the questionnaire were asked when they first received medical attention from a kidney specialist before starting dialysis. In a similar fashion, respondents to the questionnaire were asked about the number of visits they had with a nephrologist during the year and about education concerning dialysis and diet before the start of ESRD.

### Statistical Analysis

Univariate analyses of the differences in the clinical and laboratory variables between ER and LR were performed using the *t* test for continuous variables and the chi-square or Fisher exact test for discrete variables. The multivariate Cox proportional hazards regression models were used to explore the relationships of each of the independent factors with mortality risk in ESRD patients. A stepwise selection process was used to develop the multivariate Cox proportional hazards regression model. Model 1 was built to explore the relationship of timing of referral with mortality risk without the use of any explanatory variables. Model 2 was only controlled for age and gender. Model 3 was adjusted for body mass index (BMI), modified CCI, serum calcium, high-density lipoprotein cholesterol, triglycerides, total cholesterol, hemoglobin, intact parathyroid hormone, uric acid, and eGFR. The effects of the multivariate Cox proportional hazards regression models are shown as hazard ratio (HR) and 95% confidence index (CI). The Kaplan-Meier method was used to compare survival curves, and differences were assessed by means of the log rank test. The statistical analysis was performed using SAS version 9.2 (SAS Institute Inc., Cary, NC, USA) and R 2.14.1. Significant differences were defined as P less than 0.05.

## Results

### Patient Characteristics by Referral Pattern

Of 1028 patients enrolled in the CRC ESRD, 599 were referred early and 429 were referred late. Patients' clinical and laboratory characteristics are listed in Table 1. Time from referral to dialysis was significantly longer in the ER group compared to the LR group (62.3±58.9 months versus 2.9±3.4 months, P<0.001). The most common etiology of kidney failure was diabetic nephropathy in both groups. At the time of referral to a nephrologist, blood pressure (BP) was lower, renal function was better, and hemoglobin level was higher in the ER group compared to the LR group. However, at the time of dialysis, most findings including co-morbidity index were similar in both groups, except for BP, serum phosphate, total cholesterol, and low-density lipoprotein (LDL) cholesterol. Hemoglobin level or iron status was the same for the two groups. BP was well controlled in the ER group. Serum phosphate, total cholesterol, and LDL cholesterol levels in the ER group were significantly lower than for the LR group.

**Table 1:** Patient characteristics by referral pattern.^a^

	Total ESRD				DM ESRD						
	Total (*N* = 1028)	Early referral (*N* = 599)	Late referral (*N* = 429)	P Value	Total (*N* = 511)		Early referral (*N* = 302)	Late referral (*N* = 209)		P Value	
Age at the time of dialysis (year)	57.0±13.9	57.4±13.5	56.5±14.4	0.329	59.1±11.5		59.9±11.4	57.8±11.5		0.044	
Gender (male, %)	59.6	58.3	61.5	0.303	63.0		60.6	66.5		0.174	
Length of follow-up (mo)	10.2±6.9	10.7±7.1	9.4±6.6	0.003	10.1±6.8		10.8±7.3	9.1±5.9		0.004	
Drop out (N, %)	95 (9.2)	49 (8.2)	46 (10.7)	0.190	46 (9.0)		24 (7.9)	22 (10.5)		0.347	
Findings at the time of referral to nephrologist											
Underlying kidney disease (%)				0.181							
Diabetes mellitus	49.7	50.4	48.7								
Hypertension	17.5	15.5	20.3								
Glomerulonephritis	15.3	18.0	11.4								
Polycystic kidney disease	2.0	2.8	0.9								
Others	7.8	8.0	7.5								
Unknown	7.7	5.2	11.2								
Systolic BP (mmHg)	147.1±27.6	143.3±26.8	151.0±28.0	<0.001		146.0±26.2	143.8±26.2	148.7±26.0		0.086	
Diastolic BP (mmHg)	84.7±16.5	83.4±16.0	86.1±16.9	0.032		81.3±14.8	80.5±15.1	82.2±14.5		0.311	
Serum creatinine (mg/dL)	4.8±4.1	2.6±1.5	7.2±4.6	<0.001		4.1±3.0	2.6±1.4	6.0±3.4		<0.001	
eGFR (mL/min/1.73m^2^)	24.5±33.9	36.1±42.7	11.4±8.0	<0.001		24.5±21.3	34.2±24.0	12.8±8.2		<0.001	
Hemoglobin (g/dL)	10.0±5.0	11.2±6.4	8.8±1.8	<0.001		9.7±1.9	10.5±1.8	8.9±1.7		<0.001	
Number of visits to nephrologist from referral to dialysis (%)				<0.001						<0.001	
None	8.6	0	20.5			6.1	0	14.8			
1 time	7.4	1.8	15.2			6.7	2.3	12.9			
2 times or more	84.0	98.2	64.3			87.2	97.7	72.2			
Time from referral to dialysis (month)	37.7±53.8	62.3±58.9	2.9±3.4	<0.001		29.1±37.0	46.8±39.1	3.3±3.5		<0.001	
Findings at the time of dialysis											
Modified Charlson co-morbidity index	5.1±2.5	5.2±2.5	5.0±2.5	0.218		6.1±2.2	6.2±2.3	6.0±2.1		0.189	
Davies co-morbidity index	1.0±0.9	1.0±0.9	1.0±1.0	0.919		1.5±0.8	1.5±0.8	1.6±0.8		0.656	
Serum creatinine (mg/dL)	8.4±3.9	8.3±3.6	8.5±4.2	0.147		7.8±3.1	7.8±3.1	7.7±3.2		0.642	
eGFR (mL/min/1.73m^2^)	7.6±3.4	7.5±3.2	7.7±3.7	0.288		8.1±3.4	7.9±3.2	8.4±3.6		0.115	
Hemoglobin (g/dL)	9.0±3.5	8.9±1.6	8.8±1.7	0.468		9.1±3.8	8.9±1.5	8.9±1.6	0.854		
Transferrin saturation (%)	33.3±51.8	33.0±46.2	33.8±58.6	0.825		32.4±62.3	30.9±44.6	34.5±81.2		0.553	
Hemoglobin A1c (%)						6.9±2.5	6.9±1.7	6.6±1.3		0.039	
Uric acid (mg/dL)	8.2±5.1	8.0±2.5	8.2±2.6	0.228		8.2±5.5	8.0±2.4	7.9±2.3		0.850	
Calcium (mg/dL)	7.9±3.9	7.8±1.0	7.7±1.1	0.331		8.0±4.2	7.8±0.9	7.7±0.9		0.148	
Phosphate (mg/dL)	5.5±3.0	5.3±1.8	5.6±2.0	0.042		5.5±2.8	5.3±1.7	5.5±1.8		0.437	
iPTH (pg/mL)	256.0±239.9	267.2±258.0	240.0±210.9	0.105		219.0±189.5	218.1±174.8	220.4±210.4		0.905	
Systolic BP (mmHg)	140.3±23.7	138.2±23.8	143.2±23.3	0.001		141.8±24.5	139.8±24.5	144.9±24.1		0.023	
Diastolic BP (mmHg)	78.4±14.1	77.3±13.2	80.1±15.0	0.002		76.7±13.1	75.4±12.4	78.5±13.4		0.008	
BMI (kg/m^2^)	23.3±6.3	23.1±3.3	23.6±8.9	0.211		23.5±3.4	23.6±3.3	23.4±3.6		0.625	
Total cholesterol (mg/dL)	159.2±50.7	156.2±48.5	163.4±53.2	0.030		157.7±55.7	153.5±51.1	163.9±61.4		0.053	
Triglycerides (mg/dL)	129.8±82.4	129.7±86.6	129.8±76.3	0.991		135.9±86.4	134.0±85.2	138.5±88.3		0.593	
LDL cholesterol (mg/dL)	93.6±55.1	90.0±53.7	98.5±56.6	0.038		93.7±66.9	89.1±66.0	100.0±67.8		0.117	
HDL cholesterol (mg/dL)	39.7±14.3	39.7±14.6	39.8±13.9	0.902		38.3±13.2	37.6±12.3	39.5±14.3		0.149	

a Values for continuous variables are means ± standard deviation; values for categorical variables expressed as proportion. BP, blood pressure; eGFR, estimated glomerular filtration rate; iPTH, intact parathyroid hormone; BMI, body mass index; LDL, low-density lipoprotein; HDL, high-density lipoprotein.

There were no significant differences in clinical and laboratory characteristics at the time of dialysis between the ER group and the LR group with diabetes nephropathy as a cause of ESRD (DM ESRD), except in terms of BP and hemoglobin A1c.

**Figure 1:**
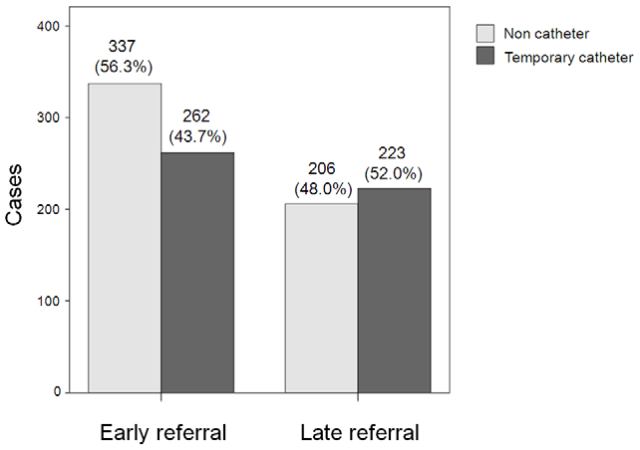
Pattern of emergency dialysis using a temporary vascular catheter according to the timing of referral. Early referral were defined as the patient's first encounter with a nephrologist occurring more than 1 year before first dialysis, with education about dialysis prior to initiation of dialysis, and all others were considered late referral (P = 0.009).

Emergency HD using a temporary vascular catheter was required in 262 of 599 ER patients compared to 223 of 429 LR patients. The rate of emergency HD in ER patients was significantly lower than in LR patients (Figure 1, 43.7% versus 52.0%, P = 0.009).

### Improved Survival in Early Referral Patients with End-stage Renal Disease

In the univariate analysis, late referral was significantly associated with patient mortality (HR 2.16, 95% CI 1.32–3.53, P = 0.002). Age (HR 1.07, 95% CI 1.04–1.09, P<0.001), hospitalization (HR 2.75, 95% CI 1.66–4.56, P<0.001), modified CCI (HR 1.33, 95% CI 1.22–1.46, P<0.001), DCI (HR 1.65, 95% CI 1.32–2.06, P<0.001), and eGFR (HR 1.13, 95% CI 1.06–1.20, P<0.001) were also significant risk factors associated with increased risk of death (Table 2). Variables that proved significant in the univariate analysis and referral pattern were included in the multivariate Cox proportional hazards model to determine factors associated with mortality. In the multivariate Cox analysis, late referral (HR 2.38, 95% CI 1.27–4.45, P = 0.007) and age (HR 1.06, 95% CI 1.03–1.09, P<0.001) had an adverse association with survival (Table 3).

**Table 2:** Univariate analysis of predictors associated with mortality.^a^

	Total ESRD (*N* = 1028)				DM ESRD (*N* = 511)							
Variable	HR	95% CI	P Value		HR		95% CI		P Value			
Age at the time of dialysis (per year increase)	1.07	1.04–1.09	<0.001		1.08		1.05–1.12		<0.001			
Gender (female)	0.95	0.58–1.57	0.851		1.23		0.65–2.35		0.523			
Underlying kidney disease												
Diabetes mellitus	Ref											
Hypertension	0.56	0.25–1.25		0.154								
Glomerulonephritis	0.63	0.30–1.30		0.207								
Polycystic kidney disease	0.59	0.08–4.27		0.598								
Others	0.70	0.25–1.96		0.497								
Unknown	1.17	0.49–2.77		0.727								
Number of visits to nephrologist from referral to dialysis												
None	Ref				Ref							
1 time	0.79	0.28–2.27	0.668		0.71		0.14–3.52		0.675			
2 times or more	0.52	0.23–1.15	0.104		0.43		0.13–1.41		0.163			
Late referral (ref = Early referral)	2.16	1.32–3.53	0.002		2.42		1.26–4.64		0.008			
Emergency HD (ref = no)	1.63	1.00–2.67	0.053		1.08		0.57–2.04		0.812			
Hospitalization (ref = no)	2.75	1.66–4.56	<0.001		2.07		1.05–4.07		0.035			
Findings at the time of dialysis												
Modified Charlson co-morbidity index	1.33	1.22–1.46	<0.001		1.26		1.10–1.43		0.001			
Davies co-morbidity index	1.65	1.32–2.06	<0.001		1.44		1.00–2.07		0.052			
eGFR (mL/min/1.73m^2^)	1.13	1.06–1.20	<0.001		1.08		0.99–1.17		0.090			
Hemoglobin (g/dL)	1.02	0.88–1.18	0.775		0.93		0.75–1.15		0.505			
Transferrin saturation (%)	1.00	0.99–1.01	0.692		1.00		0.99–1.01		0.865			
Hemoglobin A1c (%)					0.99		0.77–1.27		0.922			
Uric acid (mg/dL)	0.95	0.86–1.06	0.365		1.01		0.88–1.15		0.891			
Calcium (mg/dL)	1.11	0.88–1.40	0.392		0.91		0.65–1.27		0.571			
Phosphate (mg/dL)	0.82	0.71–0.95	0.007		0.82		0.67–1.01		0.060			
iPTH (pg/mL)	1.00	0.99–1.00	0.020		1.00		0.99–1.00		0.883			
Systolic BP (mmHg)	1.00	0.99–1.01	0.958		1.00		0.98–1.01		0.620			
Diastolic BP (mmHg)	0.98	0.97–1.00	0.082		1.00		0.97–1.02		0.915			
BMI (kg/m^2^)	0.92	0.85–1.00	0.046		0.84		0.75–0.95		0.004			
Total cholesterol (mg/dL)	1.00	0.99–1.00	0.192		1.00		0.99–1.00		0.386			
Triglycerides (mg/dL)	1.00	0.99–1.00	0.568		1.00		0.99–1.00		0.456			
LDL cholesterol (mg/dL)	0.99	0.99–1.00	0.146		0.99		0.98–1.00		0.231			
HDL cholesterol (mg/dL)	0.99	0.97–1.01	0.438		0.99		0.97–1.02		0.684			

a HR, hazard ratio; CI, confidence interval; BP, blood pressure; HD, hemodialysis; eGFR, estimated glomerular filtration rate; iPTH, intact parathyroid hormone; BMI, body mass index; LDL, low-density lipoprotein; HDL, high-density lipoprotein.

**Table 3:** Multivariate Cox proportional hazard models of independent factors of survival.^a^

Total patients (*N* = 1028)	Model 1		Model 2		Model 3	
	HR (95% CI)	P Value	HR (95% CI)	P Value	HR (95% CI)	P Value
Type of referral						
Early referral	Ref		Ref		Ref	
Late referral	2.16 (1.32–3.53)	0.002	2.28 (1.39–3.74)	0.001	2.38 (1.27–4.45)	0.007
Age (years)			3.73 (2.43–5.73)	<0.001	1.06 (1.03–1.09)	<0.001
Gender (female)			0.87 (0.53–1.44)	0.599	1.11 (0.56–2.21)	0.760
BMI					0.95 (0.86–1.05)	0.342
Calcium					1.00 (0.93–1.08)	0.964
Modified Charlson co-morbidity Index					1.09 (0.93–1.28)	0.268
HDL cholesterol					1.00 (0.97–1.03)	0.887
Triglycerides					1.00 (1.00–1.01)	0.822
Total cholesterol					1.00 (1.00–1.01)	0.326
Hemoglobin					0.90 (0.73–1.10)	0.302
eGFR					1.03 (0.94–1.13)	0.504
iPTH					1.00 (1.00–1.00)	0.163
Uric acid					1.02 (0.91–1.14)	0.794
SBP at the time of dialysis					1.00 (0.98–1.01)	0.744

DM ESRD patients (*N* = 511)	Model 1		Model 2		Model 3	
	HR (95% CI)	P Value	HR (95% CI)	P Value	HR (95% CI)	P Value
Type of referral						
Early referral	Ref		Ref		Ref	
Late referral	2.42 (1.26–4.64)	0.008	2.88 (1.49–5.59)	0.002	4.74 (1.73–13.00)	0.002
Age (years)			3.71 (2.17–6.34)	<0.001	1.08 (1.03–1.13)	0.002
Gender (female)			1.30 (0.67–2.52)	0.437	1.90 (0.67–5.38)	0.230
BMI					0.83 (0.70–0.99)	0.042
Calcium					1.00 (0.81–1.22)	0.989
Modified Charlson co-morbidity Index					1.10 (0.87–1.39)	0.440
HDL cholesterol					1.00 (0.95–1.04)	0.843
Triglycerides					1.00 (0.99–1.01)	0.640
Total cholesterol					1.01 (0.99–1.02)	0.331
Hemoglobin					0.67 (0.50–0.91)	0.010
eGFR					1.03 (0.89–1.18)	0.713
iPTH					1.00 (1.00–1.00)	0.162
Uric acid					1.03 (0.87–1.22)	0.727
SBP at the time of dialysis					0.99 (0.98–1.01)	0.341

a HR, hazard ratio; CI, confidence interval; BMI, body mass index; HDL, high-density lipoprotein; eGFR, estimated glomerular filtration rate; iPTH, intact parathyroid hormone; SBP, systolic blood pressure.

**Figure 2:**
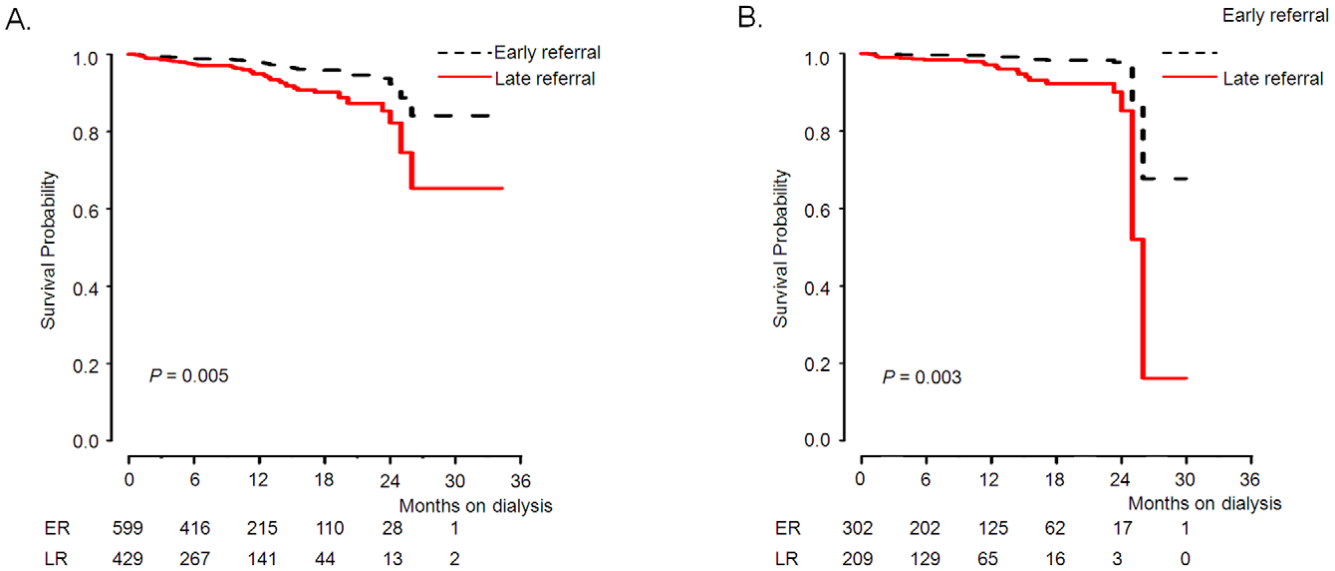
Kaplan-Meier survival curve by timing of referral. (A) Total patients, adjusted for age, gender, modified CCI, BMI, eGFR, serum hemoglobin, calcium, iPTH, uric acid, triglycerides, total cholesterol, and HDL cholesterol. (B) DM ESRD patients, adjusted for age, gender, modified CCI, BMI, eGFR, serum hemoglobin, calcium, iPTH, uric acid, triglycerides, total cholesterol, and HDL cholesterol.

In the Kaplan-Meier analysis, the survival rate in ER patients was significantly better than that in LR patients after adjusting for several risk factors of model 3 (Figure 2A, P = 0.005). The 1-year and 2-year survival rates in ER patients were 97.2% and 95.7%, respectively, compared to 93.9%, and 91.3% in LR patients.

Next, we performed a subgroup analysis in the DM ESRD patients. In DM ESRD patients, age (HR 1.08, 95% CI 1.05–1.12, P<0.001), late referral (HR 2.42, 95% CI 1.26–4.64, P = 0.008), hospitalization (HR 2.07, 95% CI 1.05–4.07, P = 0.035), and modified CCI (HR 1.26, 95% CI 1.10–1.43, P = 0.001) were the univariate factors associated with increased risk of death. In the multivariate Cox analysis, late referral (HR 4.74, 95% CI 1.73–13.00, P = 0.002) and age (HR 1.08, 95% CI 1.03–1.13, P = 0.002) had an adverse association with survival (Table 3). The survival rate in ER patients was significantly higher than that in LR patients (Figure 2B, P = 0.003).

During follow-up, 66 patients (6%) died overall. Causes of death are listed in Table 4. Cardiovascular death represented the most common cause of death (32%), followed by infection (30%), unknown cause (17%), and neoplasm (8%). The remaining deaths (13%) were due to other causes (Table 4). In LR, cardiovascular death was the most common cause of death. However, infection was the most common cause of death in ER. In the Kaplan-Meier analysis, the cardiovascular death free survival rate was significantly worse in LR patients compared with ER patients in total (P = 0.004) and DM ESRD (P = 0.008). In the multivariate Cox analysis, LR (Table S1, HR 4.99, 95% CI 1.48–16.82, P = 0.009) was significant risk factors associated with cardiovascular death. Among patients with DM ESRD, LR was still significant factor associated with increased risk of cardiovascular death (Table S1, HR 26.71, 95% CI 1.49–478.99, P = 0.026).

**Table 4:** Cause of death in patients.

		Total			DM ESRD		
		Early referral (*N*)	Late referral (*N*)	Sum (*N*)	Early referral (*N*)	Late referral (*N*)	Sum (*N*)
Cardiovascular	Myocardial infarction	0	2	2	0	2	2
disease	Cardiomyopathy	0	1	1	0	0	0
	Cardiac arrest, cause unknown	4	6	10	2	3	5
	Pulmonary edema	0	2	2	0	1	1
	Pulmonary embolus	0	1	1	0	0	0
	Cerebrovascular accident including intracranial hemorrhage	1	0	1	1	0	1
	Hemorrhage from ruptured vascular aneurysm	1	1	2	0	0	0
	Other hemorrhage	0	1	1	0	1	1
	Mesenteric infarction/ischemic bowel	0	1	1	0	1	1
Infection	Peritoneal access infectious complication, bacterial	0	1	1	0	0	0
	Peritoneal access infectious complication, fungal	0	1	1	0	1	1
	Peritonitis (complication of peritoneal dialysis)	0	1	1	0	0	0
	Septicemia, other	4	2	6	1	2	3
	Cardiac infection (endocarditis)	0	1	1	0	1	1
	Pulmonary infection (pneumonia, influenza)	4	6	10	2	4	6
Liver disease	Liver failure, cause unknown or other	0	1	1	0	0	0
Gastro-intestinal disease	Perforation of bowel	1	0	1	0	0	0
Other	Cachexia/failure to thrive	1	0	1	1	0	1
	Malignant disease, patient on immunosuppressive therapy	2	0	2	1	0	1
	Malignant disease	0	3	3	0	1	1
	Hyperkalemia	1	0	1	1	0	1
	Suicide	0	1	1	0	1	1
	Other cause of death	2	2	4	2	1	3
	Unknown	5	6	11	3	4	7
Sum		26	40	66	14	23	37

Hospitalization-free survival rate and cardiovascular event-free survival rates were not different between ER and LR patients (data not shown).

### Relationship between Mortality and Age at the Time of Referral

**Figure 3:**
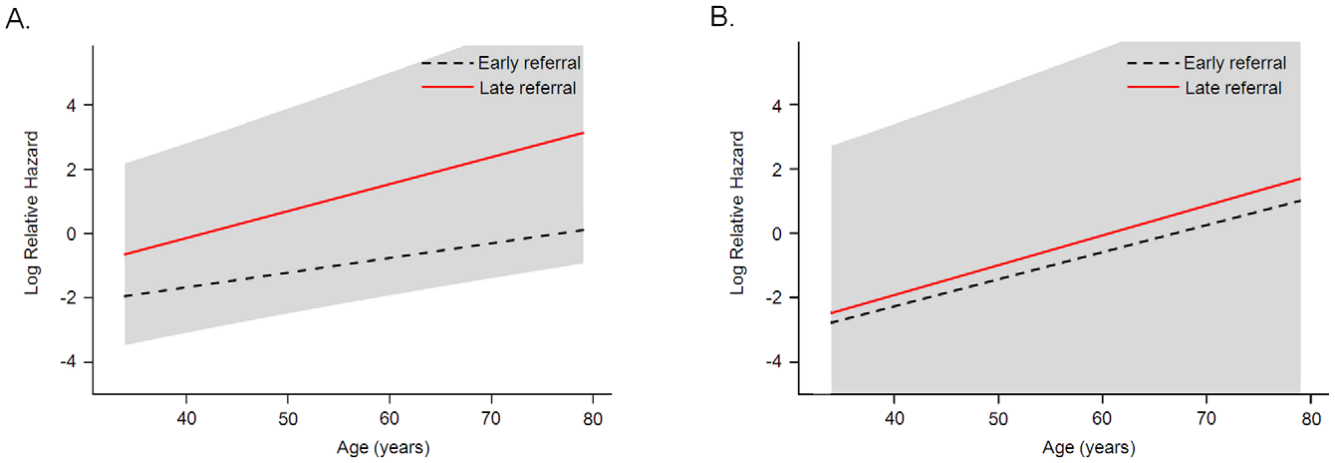
Relationship between log relative hazard for mortality and age according to timing of referral. (A) Total patients, adjusted for age, gender, modified CCI, BMI, eGFR, serum hemoglobin, calcium, iPTH, uric acid, triglycerides, total cholesterol, and HDL cholesterol. (B) DM ESRD patients, adjusted for age, gender, modified CCI, BMI, eGFR, serum hemoglobin, calcium, iPTH, uric acid, triglycerides, total cholesterol, and HDL cholesterol.

The relationship between age and mortality was evaluated. As the age of patients increased, the hazard ratio for death also increased (Figure 3A). In particular, in older patients the hazard ratios for ER and LR patients were quite different, with survival rates for ER patients increasing against LR patients. This trend was observed in DM ESRD patients (Figure 3B).

## Discussion

The present nationwide multi-center prospective cohort study showed that early nephrology referral in the predialytic stage of CKD improves survival rate in ESRD. Late referral remains an important predictor of mortality even after adjusting for age, gender, co-morbidity, BMI, and biochemical variables.

Previous studies on the association of the timing of referral to a nephrologist with mortality have demonstrated conflicting results. Kazmi *et al*. studied 2,195 patients between 1996 and 1997 and found that, compared with ER patients, LR (<4 months) patients had a 44% higher risk of death at 1 year after initiation of dialysis, which remained significant after adjusting for the quintile of the propensity score [11]. Dogan *et al* studied 101 patients between 1998 and 2002 and found that ER (>12 weeks) and/or early diagnosis of ESRD resulted in better biochemical variables, shorter length of first hospitalization, a higher percentage of elective construction of arteriovenous fistula, and the availability to start with an alternative dialysis modality [12]. In contrast, two studies have shown no difference in mortality between ER and LR patients. Schmidt *et al* showed that there was no statistically significant difference in long-term survival when ER (>1 month) patients were compared with LR patients at 4 months among 238 patients [13]. Roubicek *et al* showed that the referral pattern (LR <4 months) was not associated with mortality rate among 270 patients [24].

Many previous studies incorporated an observational retrospective design, and the definition of the timing of ER was different in each study. In addition, some prospective studies were of small size and/or single-center design. Furthermore, the referral timing of 3 months or more in most previous studies was too short to assess and educate patients.

In a recent study, Di Napoli *et al* reported that late referral patients had a lower frequency of hepatitis B virus vaccination, arteriovenous fistula and information about renal replacement therapy modalities, and they more often started chronic dialysis in an emergency [18]. They defined LR patients as those who had not been regularly referred to a nephrologist in the one year before chronic dialysis began. And, they considered a period of 12 months as adequate to describe the role of individual and health service characteristics in early access to renal services for ESRD care. In addition, de Jager DJ *et al* classified referral (time between first pre-dialysis visit to a nephrologist and dialysis initiation) as: late (<3 months), early (3–12 months) or very early (≥12 months). They reported that early and late referrals were associated with increased mortality compared with very early referral [25]. Quaglia M *et al* also mentioned that predialysis nephrology care had a much wider concept than providing the patient with a dialysis access and consequently demanded a longer time (ie, several years) to produce results, and that 3-month period before dialysis was inadequate to assess any impacts on hard end points [17].

This study is a prospective nationwide multi-center cohort study. ER was defined as a group undergoing follow-up for 12 months or longer prior to the initiation of dialysis. And, a 1-year period before dialysis was enough to control a BP, metabolic condition of patients, and provided the predialysis care for patients. Thus, this study design provides for a proper evaluation of the association between referral pattern and mortality in ESRD patients.

Our study showed that the mortality rate increased by 2.4 times during overall follow-up and this risk remained after adjusting for age, gender, and other covariates. Furthermore, we analyzed the cause of death in ESRD patients according to referral pattern. There were few researches studied about association between referral pattern and causes of death in dialysis patients. Lorenzo *et al* reported that referral pattern was no significant association with causes of death in dialysis patients [15]. However, Herget-Rosenthal *et al* showed that cardiovascular death was increased in LR versus ER (patient number, 4 versus 1) [26]. In this study, we showed the data that cardiovascular death was increased in LR versus ER, and the cardiovascular death free survival rate in ER was significantly better than that in LR. Blood pressure was well controlled in the early referral (ER) group. However, ER significantly lowered all-cause mortality and cardiovascular mortality after adjusting for systolic blood pressure in the multivariate analysis. Recently, Winkelmayer *et al* reported that estimated annual reductions in 1-year mortality rates were 0.9% (95% CI, 0.7%–1.1%) in ER (>90 days), and there was no material improvement in 1-year survival rates after dialysis initiation among 323,977 patients [5]. But, considering the duration of patients' referrals to a nephrologist, the timing of early referral (>90 days) was a short period in which to evaluate and educate ESRD patients. In the present study, with increasing age, the risk for mortality due to late referral increased in the total patient population. In particular, in older patients there was a big difference in the hazard ratio between ER and LR (<1 year) patients.

It has been reported that patient education by nephrologists before initiation of dialysis decreases the likelihood of the need for urgent dialysis, resulting in a reduction in the need for the creation of a temporary vascular access [27]. Schmidt *et al* showed that the need for emergency HD was significantly less among ER patients compared with LR patients (22% versus 90%) [13]. Our analysis also found that the likelihood of patients referred early receiving emergency HD using a temporary vascular catheter was significantly reduced. A previous study has shown that reduced use of a temporary vascular catheter is associated with decreased patient morbidity and mortality through reduced systemic infection rates [28].

Patients who are referred earlier are prepared for dialysis initiation, resulting in fewer emergent hospitalizations. Smart *et al* showed a reduction of 8.8 days (95% CI, -10.7 to -7.0 days, P<0.00001) in those referred earlier to a nephrologist [29]. However, the hospitalization-free survival rate was no different for ER and LR patients in our study.

In this study, ER patients were well educated about dialysis by a nurse or nephrologist. Furthermore, BP, serum phosphate, total cholesterol, and LDL cholesterol were well controlled in the ER group. This could reduce the cardiovascular mortality rate and all-cause mortality in ER patients. Thus, timely referral to a nephrologist at least 1 year before dialysis initiation is important to reduce mortality even in older patients. Predialysis nephrology care is a much wider concept than providing the patient with dialysis access. It is important for the nephrologist to give patients information about CKD and offer personal education about dialysis or lifestyle modification. ER provides identification and correction of reversible causes of CKD, and preparation of dialysis. Timely referral is expected to influence long-term survival in ESRD patients.

Our results are informative, but this study has a limitation. This study had a relatively short follow-up period, with a maximum of 36 months. This was a short period in which to analyze the survival in ESRD patients.

In conclusion, timely referral to a nephrologist in the predialytic stage is associated with reduced mortality.

## Supporting Information

Table S1 Multivariate Cox proportional hazard models of independent factors of cardiovascular free survival.
(DOC)
