# Supplementary material for: Early Referral to a Nephrologist Improved Patient Survival: Prospective Cohort Study for End-Stage Renal Disease in Korea
Source: PLoS One. 2013 Jan 25;8(1):e55323. doi: 10.1371/journal.pone.0055323 (PMC3555934; doi:10.1371/journal.pone.0055323)
Supplement: Table S1 — Multivariate Cox proportional hazard models of independent factors of cardiovascular free survival. (DOC) [file pone.0055323.s001.doc]

**Table S1. Multivariate Cox proportional hazard models of independent factors of cardiovascular free survival** ^a^

| **Total patients** (N=1028**)** | | **Model 1** | |  | **Model 2** | |  | **Model 3** | |
| --- | --- | --- | --- | --- | --- | --- | --- | --- | --- |
|  |  | HR (95% CI) | *P* Value |  | HR (95% CI) | *P* Value |  | HR (95% CI) | *P* Value |
| Types of referral | |  |  |  |  |  |  |  |  |
|  | Early referral | Ref |  |  | Ref |  |  | Ref |  |
|  | Late referral | 3.74 (1.43–9.74) | 0.007 |  | 3.93 (1.51–10.26) | 0.005 |  | 4.99 (1.48–16.82) | 0.009 |
| Age (years) | |  |  |  | 1.05 (1.01–1.09) | 0.007 |  | 1.03 (0.97–1.09) | 0.322 |
| Gender (Female) | |  |  |  | 1.19 (0.49–2.88) | 0.701 |  | 1.56 (0.47–5.25) | 0.470 |
| BMI | |  |  |  |  |  |  | 0.95 (0.79–1.13) | 0.551 |
| Calcium | |  |  |  |  |  |  | 1.00 (0.89–1.13) | 0.959 |
| Modified Charlson co-morbidity Index | |  |  |  |  |  |  | 1.16 (0.88–1.54) | 0.284 |
| HDL-cholesterol | |  |  |  |  |  |  | 1.00 (0.96–1.05) | 0.899 |
| Triglyceride | |  |  |  |  |  |  | 1.00 (1.00–1.01) | 0.173 |
| Total cholesterol | |  |  |  |  |  |  | 1.00 (0.99–1.01) | 0.939 |
| Hemoglobin | |  |  |  |  |  |  | 0.90 (0.62–1.31) | 0.580 |
| eGFR | |  |  |  |  |  |  | 1.08 (0.93–1.25) | 0.314 |
| iPTH | |  |  |  |  |  |  | 1.00 (1.00–1.00) | 0.727 |
| Uric acid | |  |  |  |  |  |  | 0.96 (0.77–1.20) | 0.713 |
| SBP at the time of dialysis | |  |  |  |  |  |  | 1.00 (0.98 – 1.03) | 0.917 |
| **DM ESRD patients** (N=511) | | **Model 1** | |  | **Model 2** | |  | **Model 3** | |
|  |  | HR (95% CI) | *P* Value |  | HR (95% CI) | *P* Value |  | HR (95% CI) | *P* Value |
| Types of referral | |  |  |  |  |  |  |  |  |
|  | Early referral | Ref |  |  | Ref |  |  | Ref |  |
|  | Late referral | 5.32 (1.37–20.65) | 0.016 |  | 7.04 (1.75–28.42) | 0.006 |  | 26.71 (1.49–478.99) | 0.026 |
| Age (years) | |  |  |  | 1.07 (1.01–1.14) | 0.022 |  | 1.13 (0.97–1.31) | 0.108 |
| Gender (Female) | |  |  |  | 2.47 (0.73–8.33) | 0.146 |  | 0.84 (0.50–14.17) | 0.906 |
| BMI | |  |  |  |  |  |  | 0.43 (0.20–0.91) | 0.029 |
| Calcium | |  |  |  |  |  |  | 1.05 (0.86–1.28) | 0.634 |
| Modified Charlson co-morbidity Index | |  |  |  |  |  |  | 0.86 (0.39–1.88) | 0.698 |
| HDL | |  |  |  |  |  |  | 1.08 (0.93–1.26) | 0.298 |
| Triglyceride | |  |  |  |  |  |  | 1.01 (0.98–1.03) | 0.621 |
| Total cholesterol | |  |  |  |  |  |  | 1.02 (0.98–1.06) | 0.337 |
| Hemoglobin | |  |  |  |  |  |  | 0.53 (0.22–1.29) | 0.161 |
| eGFR | |  |  |  |  |  |  | 1.13 (0.74–1.74) | 0.566 |
| iPTH | |  |  |  |  |  |  | 1.01 (1.00–1.02) | 0.015 |
| Uric acid | |  |  |  |  |  |  | 1.22 (0.85–1.76) | 0.290 |
| SBP at the time of dialysis | |  |  |  |  |  |  | 1.00 (0.94–1.06) | 0.981 |

^a^HR, hazard ratio; CI, confidence interval; BMI, body mass index; HDL, high-density lipoprotein; eGFR, estimated glomerular filtration rate; iPTH, intact parathyroid hormone; SBP, systolic blood pressure.
